# The Diphtheria Cure

**Published:** 1895-03

**Authors:** 


### The Diphtheria Cure.

The matter of the control or supervision of the use of the anti-toxine, the new diphtheria cure, is engaging the attention of the local authorities in Berlin, and Dr. Kinyoun, of the Marine Hospital Service, reports that on November 4th, Professor Koch convened a meeting of the Prussian board of health for determining what action should be taken in that regard. Professor Koch had expressed the opinion that there should be some government supervision of the serum, so that it could always be relied upon. If there was no such supervision, it would not be very long before spurious articles would be put on the market, and not only a good remedy would be brought into disrepute, but lives would be sacrificed when they might be saved.

It was decided at the meeting of the board that all serum intended for use in Prussia should be inspected and tested for its purity and strength before it would be allowed to be used. This step, the doctor reports, was satisfactory to all parties concerned, and will be the means of insuring a good article of standard strength at all times for Prussia.

In this connection Dr. Kinyoun calls attention to what will evidently occur in our own country. Many persons will, during the coming year, commence to prepare the serum as a business enterprise, and there will, without doubt, be many worthless articles called anti-toxine thrown upon the market. All the serum intended for sale, he believes, should be made or tested by competent persons. The testing, in fact, should be done by disinterested parties.

The anti-toxine, says the report of Dr. Kinyoun, never will work miracles. It has its limits like any other agent, and like a perfect piece of machinery will not accomplish the full result unless directed by a skilled hand. "Some persons affected with this dread disease will succumb, it matters not how soon we apply the remedy. The majority will, however, I am sure, recover if the anti-toxine is given early and properly."

In closing, the report expresses the hope that soon every State and municipality will take the proper steps to provide facilities for supplying the remedy to the people.

Incorporated in Dr. Kinyoun's report are a number of tables or charts, showing the effects of the respiration, pulse and temperature of the administration of the anti-toxine in various cases.

Dr. Kinyoun discussed anti-toxine before the Washington Medical Society. A reporter says:

"The life-destroying effects of diphtheria need no longer be dreaded. Dr. Joseph J. Kinyoun, of the United States Marine Hospital Service, as the representative of this government, attended the recent Medical Congress in Paris. While absent the doctor made a thorough study of the anti-toxine treatment and brought home with him a quantity of the serum.

"Before the District Medical Society last night Dr. Kinyoun read a paper on the 'Anti-toxine Treatment for Diphtheria.' He described the successful tests of the inoculation of the serum, and maintained that the efficacy of the treatment had been established beyond question. The doctor said he was in favor of governmental or municipal control, or at least supervision, of the mauufacture of the anti-toxine to insure its being of standard quality."—*Journal H. S. and C., of S. S.*, February.

[By all means the manufacture and sale of this important preparation should be under medical supervision and control, else the very name will soon become a reproach. Texas should have facilities for its preparation, but we can not just now hope for any sanitary legislation that is likely to cost the State anything;—legislators have "retrench" on the brain. We will have to look to the M. H. S., for our supply I suppose, for the present.]

---

**Dr. Charles A. L. Reed,** of Cincinnati, announces the removal, during March, of his private hospital for abdominal and pelvic surgery, from 487 West Sixth St., to his recently acquired property in St. Leger Place, Walnut Hills. The situation is salubrious, quiet, high, and possesses a commanding view, while the hospital itself fulfills all modern requirements.

---

**His Idea of Luxury.**—"It seems to me maw has a mighty easy time," sniffed Johnny, who was shoveling off the sidewalks in the back yard for the third time since breakfast. "She hain't nuthin to do but stay in the house all day and doctor her neuralgia."

